# Graves’ disease overlapping with chronic hepatitis B and methimazole-induced liver injury and autoimmune hepatitis: a case report

**DOI:** 10.1186/s12876-022-02133-z

**Published:** 2022-02-10

**Authors:** Meifang Zheng, Shiyuan Cui, Wei Zhang, David R. Brigstock, Runping Gao

**Affiliations:** 1 Department of Hepatic Biliary Pancreatic Medicine, First Hospital of Jilin University, 71 Xinmin Avenue, Changchun, 130021 Jilin China; 2 The Research Institute at Nationwide Children’s Hospital, Columbus, OH USA

**Keywords:** Graves’ disease, Hepatitis B virus, Glucocorticoids, Methimazole, Case report

## Abstract

**Background:**

Liver injury related to Graves’ Disease (GD) includes hepatotoxicity of thyroid hormone excess, drug-induced liver injury, and changes resulting from concomitant liver disease. Methimazole (MMI) has been shown to induce several patterns of liver injury. However, the diagnosis and treatment of autoimmune hepatitis (AIH) overlapping with either GD or chronic hepatitis B are challenging.

**Case presentation:**

A 35-year-old man from China presented with a two-year history of GD and a 10-day history of progressive jaundice. He had taken MMI for two months and discontinuing treatment due to liver toxicity 1 year ago and for another 6 days 20 days prior to hospitalization. The patient was diagnosed with GD overlapping with chronic hepatitis B and MMI-induced liver injury with early stage of acute-on-chronic liver failure on admission. However, the elevated aminotransferase and bilirubin levels could not be controlled after correction of liver failure and effective control of HBV replication and hyperthyroidism by daily oral entecavir and one-time oral administration of 131-iodine. The patient underwent liver biopsy on the 43rd day of hospitalization, showing HBsAg expression on the membrane of hepatocytes and typical histopathological characteristics of AIH. He was finally diagnosed with GD overlapping with chronic hepatitis B and MMI-induced liver injury and AIH. The elevated aminotransferase and bilirubin completely returned to normal by 3-month glucocorticoid therapy and continuous entecavir treatment and there was no recurrence during a 6-month follow-up, suggesting that AIH in this patient is different from classical AIH or GD-associated AIH.

**Conclusions:**

GD together with AIH is a complex and difficult subject. It needs to be clarified whether MMI or HBV can act as a trigger for AIH in this patient.

## Background

Liver dysfunction related to hyperthyroidism includes abnormalities associated with the effects of thyroid hormone excess, drug-induced liver injury, and changes resulting from concomitant liver disease [1, 2]. Methimazole (MMI) is the first-line anti-thyroid agent used in clinical practice for management of Graves’ disease (GD) [2] which is the most common cause of hyperthyroidism [3]. MMI is also associated with a certain degree of hepatic injury, although its adverse effects are less severe than those of propylthiouracil (PTU) [1]. Clinically, the characteristics of MMI-induced hepatotoxicity are variable and include necro-inflammation, granulomas, and/or steatosis, with a dominant pattern of cholestasis [3–5].

MMI has been demonstrated to decrease adenosine triphosphate (ATP) levels, to increase reactive oxygen species (ROS) content, and to damage mitochondrial membranes in the liver of mouse models [6]. However, MMI seems to be a novel mitochondrial protecting agent in vitro, suggesting that MMI bioactivation and reactive metabolite formation potentially contribute to liver injury [6]. Recently a metabolomic study in patients with GD revealed that monoamine oxidase inhibition, ROS production, mitochondrial dysfunction, and DNA disruption might contribute to MMI-induced hepatotoxicity [7]. It has also been reported that MMI induces autoimmune diseases including antineutrophil cytoplasmic antibody (ANCA)-positive microscopic polyangiitis and insulin autoimmune syndrome (IAS) [8, 9]. Allergic reactions are considered to contribute to MMI-induced liver injury and cholestatic jaundice, and some of these cases have been successfully controlled by glucocorticoids [4, 10]. A case of GD complicated with MMI-induced liver injury with AIH histological features has been described, implying MMI may act as a trigger for AIH [11]. GD overlapping with chronic hepatitis B (CHB) is not uncommon in China. AIH superimposed on CHB has also been reported. However, there are different opinions as to whether hepatitis B virus (HBV) functions as a trigger for AIH [12, 13].

In this report, a case from Northeast China of GD overlapping with CHB and MMI-induced liver injury and AIH is described. This rare clinical case has never been reported in literature.

## Case presentation

A 35-year-old man was admitted to hospital due to progressive jaundice for 10 days. He had a two-year history of GD. MMI 30 mg/day was initiated one year ago, but was discontinued after two months due to symptoms of fatigue, palpitations and diarrhea and abnormal liver function tests (alanine aminotransferase, ALT 268 U/L, total bilirubin, TBil 54.7 μmol/L), and his liver function was normal one month later. MMI 30 mg/day was taken again for 6 days 20 days prior to hospitalization but discontinued because of same symptoms. Serum HBsAg was positive and the liver function test revealed increased levels of aspartate aminotransferase (AST, 722.3 U/L), alanine aminotransferase (ALT, 818.4 U/L), alkaline phosphatase (ALP, 192 U/L), total bilirubin (TBil, 447.7 μmol/L) in the local hospital one week before. The patient had no histories of alcohol, tobacco, hypertension and heart disease and his serum HBsAg had not been detected until one week prior to hospitalization. Physical examination on admission to this hospital showed a blood pressure of 135/78 mmHg, a heart rate of 100 bpm, intense jaundice, mild diffuse thyromegaly. The liver and spleen were not palpable. Liver function test were as follows: AST 133.9 U/L, ALT 137.7 U/L, ALP 149.3 U/L, cholinesterase (CHE) 2676 U/L, albumin (Alb) 30.4 g/L, TBil 831.9 μmol/L, direct bilirubin (DBil) 554.6 μmol/L, total bile acid (TBA) 493.5 μmol/L. Fasting blood glucose was 2.8 mmol/L. Prothrombin activity (PTA) was 38% and international normalized ratio (INR) was 1.51. Thyroid stimulating hormone (TSH) was 0.0025 μIU/mL, free triiodothyronine (FT3) was more than 30.7 pmol /L (upper limit of normal (ULN), 6.01 pmol /L), anti-thyroglobulin antibody (TgAb) was positive. Tests for anti-HAV, anti-HEV, anti-EBV, anti-CMV IgMs and anti-HCV IgG were negative. Tests for HBsAg, HBeAg and anti-HBc were positive. HBV DNA was 3.28 × 10^4^ IU/mL. The titer of the antinuclear antibody (ANA) was 1: 320. IgG was 22.17 g/L (ULN, 17.4 g/L) and IgA and IgM were within normal limits. 2 h and 24 h radioiodine uptakes were 37.9% and 41.3% respectively*.* CT showed normal liver morphology and mild splenomegaly. Ultrasound showed a diffusely enlarged thyroid. The echocardiogram was normal.

**Fig. 1:**
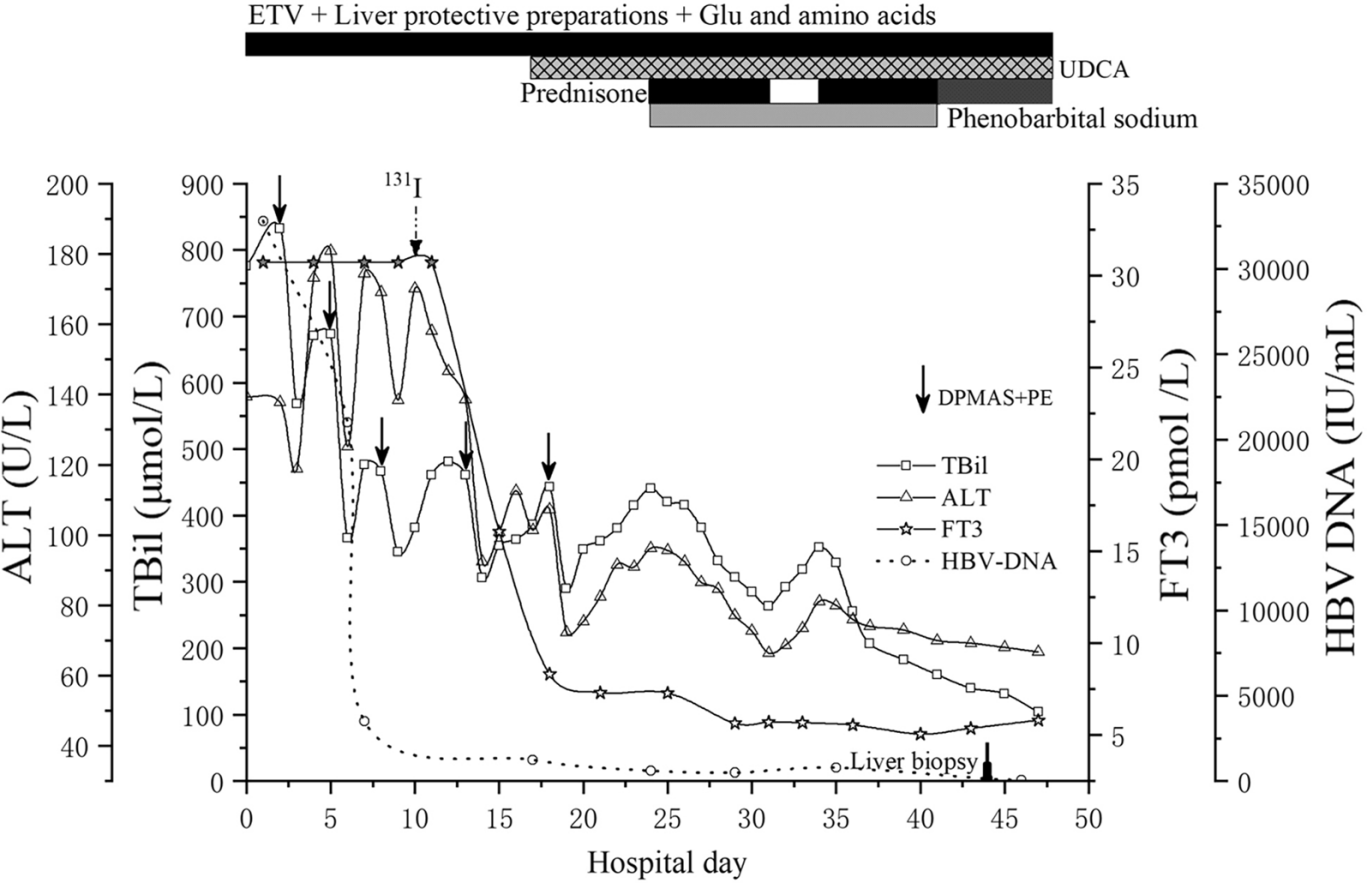
Laboratory examination results during hospitalization. *ALT* Alanine transaminase, *TBil* Total bilirubin, *FT3* Free triiodothyronine, *HBV DNA* Hepatis B virus desoxyribonucleic acid, *ETV* Entecavir, *UDCA* Ursodeoxycholic acid, *Glu* Glucose, *DPMAS* Dual plasma molecular adsorption system, *PE* Plasma exchange

**Fig. 2:**
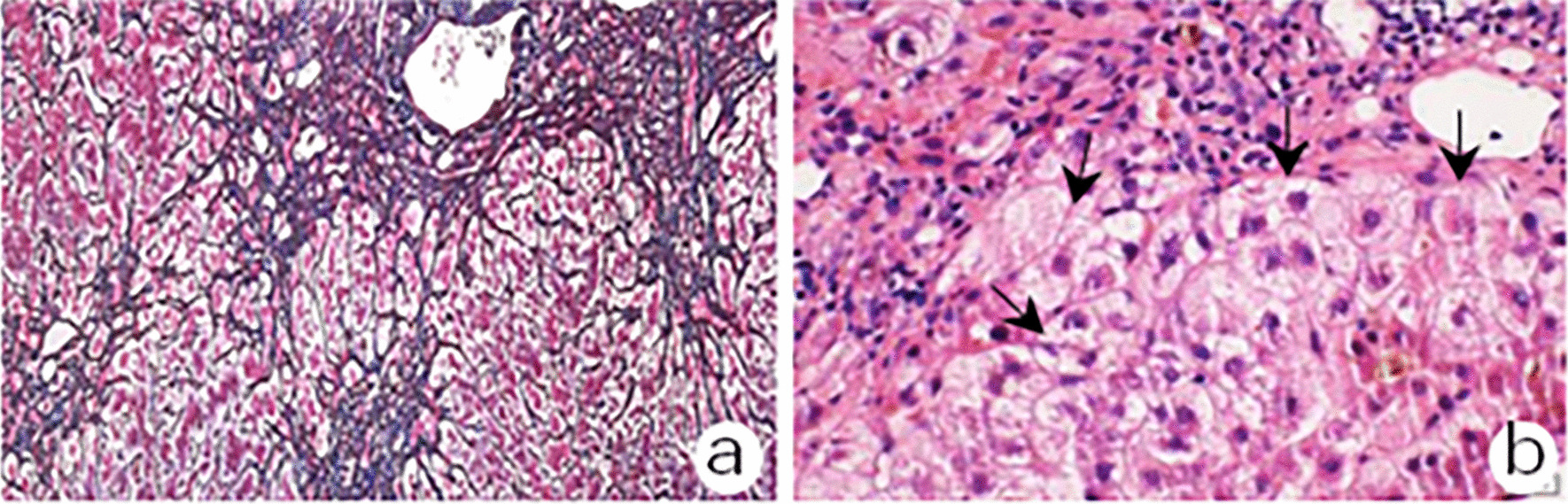
Laboratory examination results during hospitalization. **a** Masson trichrome and reticular fiber staining showed partial adjacent portal areas were connected to separate the surrounding hepatic parenchyma and mild interstitial fibrosis; **b** HE staining showed a moderate lymphoplasmacytic infiltration in the portal area, obvious interface hepatitis and hepatocyte rosettes (arrow), bile pigment granules in hepatocytes and bile plugs formation in bile capillaries

The patient was preliminarily diagnosed with GD complicated with CHB, MMI-induced liver injury and early-stage acute-on-chronic liver failure (ACLF). Entecavir (ETV) (0.5 mg/d) was used for HBV inhibition. Acetylcysteine (8 g/d) in 100 ml of 5% glucose solution and ademetionine (1 g/d) in 250 ml of 5% glucose solution were transfused for liver protection. Besides eating regularly, a 1750 ml solution including 225 g of glucose, 50 g of compound amino acids, 10 mg of vitamin B1, 5 mg of vitamin B2, 200 mg of vitamin C, 4 g of potassium chloride and 25 IU of insulin was transfused daily for 20 days against low glucose and hypermetabolism. Dual plasma molecular adsorption system (DPMAS) plus adequate plasma exchange (PE) was performed on the 2nd, 5th and 8th days of hospitalization, which significantly reduced the serum bilirubin levels. On the 10th day after admission, radioactive iodine (^131^I) was applied to treat GD. Levels of FT3 gradually decreased and coagulation function also improved; However, TBil was still at a high level (Fig. 1). Another two DPMAS plus PE therapies were performed on the 12th and 18th days after admission. Ursodeoxycholic acid (UDCA) was taken orally on the 19th day after admission, but ALT, TBil and TBA levels were progressively increased. On the 24th day after admission, laboratory examination showed ALT 96.2 U/L, TBil 440.1 μmol/L, TBA 491.8 μmol/L, PTA 67%, HBV DNA 20.4 IU/ml and FT3 7.1 pmol/L. The patient felt itchy and experienced insomnia. He began to receive prednisone 30 mg orally per day and phenobarbital sodium 50 mg intramuscularly each night. Seven days later, the ALT level was reduced to 68.4 U/L and TBil was 261.9 μmol/L. He stopped taking prednisone for 3 days upon which ALT and TBil levels rebounded instead of decreasing continuously. Prednisone administration (30 mg/d) was re-instituted and 7 days later, ALT and TBil levels had decreased and the patient felt no discomfort. Phenobarbital sodium was discontinued and he was treated with a tapering course of oral prednisone. On the 43rd day of hospitalization liver biopsy was performed. Masson trichrome and reticular fiber staining showed partial adjacent portal areas were connected and separate the surrounding hepatic parenchyma and mild interstitial fibrosis; hematoxylin–eosin (HE) staining showed a moderate lymphoplasmacytic infiltration in the portal area, obvious interface hepatitis and hepatocyte rosettes, bile pigment granules in hepatocytes, and bile plugs formation in bile capillaries (Fig. 2). Immunohistochemical staining of CK7 and HBsAg showed moderate proliferative reaction of the periportal bile duct and high expression of HBsAg on the membrane of hepatocytes (data not shown). According to the clinical features and a 7-point RUCAM score and a 6-point simplified IAIHG score (Table 1), the patient was diagnosed as GD overlapping with CHB and MMI-induced liver injury and AIH. On the 47th day of hospitalization, laboratory examination revealed AST 50.3 U/L, ALT 69.1 U/L, TBil 104.7 μmol/L, HBV DNA 10.0 IU/mL, and FT3 5.81 pmol/L (Fig. 1). The patient was discharged without discomfort and continued to take ETV (0.5 mg/d), prednisone (25 mg/d × 1 w, 20 mg/d × 2 w, 15 mg/d × 3 w, 10 mg/d × 4 w), ademetionine (1 g/d) and UDCA (500 mg/d). Liver function returned to normal 70 days after discharge, along with a normal level of IgG and ANA. The patient then received a long-term maintenance dose of ETV 0.5 mg/d after stopping prednisone and UDCA. There was no recurrence during a 6-month follow-up.

**Table 1:** Autoimmune hepatitis score using simplified scoring system

Variable	Score
ANA ≥ 1:80	2
IgG > 1.1 times ULN*	2
Liver histology: typical AIH	2
Absence of viral hepatitis: no	0
Total	6 (probable)

*Upper limit of normal

## Discussion and conclusions

CHB is a chronic inflammatory disease of the liver caused by persistent HBV infection, which shows the pathological feature of piecemeal and/or bridging necrosis, infiltration of inflammatory cells and fibrosis. CHB develops insidiously and can only be detected by laboratory examination. Acute exacerbation of chronic hepatitis B (AECHB) involves cytotoxic T cell-mediated cytolysis of HBV antigen(s)-expressing hepatocytes, manifesting as a sudden increase in HBV DNA and an abrupt elevation of ALT to > 5 ULN or a greater than threefold increase in ALT, whichever is higher [14]. Most cases of AECHB have mild symptoms, but some cases have acute hepatic decompensation or even death [14]. The histopathological characteristic of ACLF caused by AECHB shows massive or submassive liver necrosis [15]. Although the patient had not been tested for HBV previously, he had splenomegaly, an elevation of ALT (> 5 ULN), 3.28 × 10^4^ IU/mL of HBV DNA, and a decrease of PLT and CHE after admission. He also had a histopathological feature (G3S3) and an immunocytodiagnostic marker (HBsAg expressing on the membrane of hepatocytes) of CHB. Taken together these findings support a clinical diagnosis of CHB but ACLF, severe intrahepatic cholestasis, periportal bile duct reaction and typical AIH histological features are not the clinical manifestation and pathological characteristics of CHB.

Although GD can induce malnutrition, thyrotoxic hepatitis usually happens when decompensated hyperthyroidism is present [1]. In the last decade, hepatotoxicity and cholestasis induced by MMI have been reported [3, 4, 10, 15]. The mechanisms of MMI-associated liver injuries are following: (1) relative hypoxia of the portal system which easily leads to hepatocyte degeneration and necrosis; (2) MMI may aggravate jaundice in the patients with inherited disorders of bilirubin metabolism; (3) several active substances produced in the process of MMI metabolism may damage the hepatocyte and stroma; (4) an allergic reaction [4]. Glucocorticoids are used to effectively treat cholestasis induced by MMI despite the lack of histopathological evidence of the liver [4, 10]. This patient experienced twice liver injuries when he took MMI for two months one year ago and for 6 days after the second MMI medication. He had a RUCAM score of 7 [16]. Thus, the patient met the diagnostic criteria for MMI-induced liver injury.

The patient also had a simplified IAIHG score of 6 based on a typical AIH histological feature and higher levels of IgG and ANA. Additionally, the elevated ALT and bilirubin rapidly reduced to normal after 3-month glucocorticoid therapy without recurrence during a 6-month follow-up. The patient met the diagnostic criteria for AIH, but which was different from classical AIH [17, 18].

GD together with AIH is a complex and difficult subject to deal with. It contains three forms including GD-associated AIH, GD coexisting with classical AIH and GD complicated with autoimmune-like drug-induced liver injury (AI-DILI), each of which has its own clinical features (Table 2). Briefly, GD-associated AIH was extremely rare and this kind of AIH could be eliminated following a cure of hyperthyroidism [19]. The coexistence of GD with classical AIH is extremely difficult to treat. The combination treatment of long-term steroid use plus anti-thyroid drug or radioactive iodine ablation was indispensable. However, AIH still exists in the case of effective control of hyperthyroidism and the recurrence of AIH may occur if steroid therapy is to be discontinued [11, 20]. Some of patients with GD coexisting with classical AIH eventually require liver transplantation [11]. Although the pathogenesis of AI-DILI has not yet been fully elucidated, it is generally accepted that the covalent binding of a reactive drug metabolite to a hepatocyte surface protein, formation of a neoantigen, activation of CD8^+^ T cells with nonselective antigen receptors, and deficient immune regulatory mechanisms are the main bases for a transient loss of self-tolerance, thereby inducing short-term hepatocyte damage [18]. PTU, like other drugs (minocycline, fenofibrate, methyldopa, and statins, and so on), has been reported to induce AI-DILI [21, 22]. This drug has also been warned by the Food and Drug Administration (FDA) for treating GD in 2016 because of the risk of severe liver injury [2]. The liver injury of GD complicated with PTU-induced AI-DILI was recovered rapidly after one-month steroid therapy [22]. In 2019, Sano S reported a case of GD complicated with MMI-induced liver injury with AIH histological features. The elevated aminotransferase levels of the patient couldn’t be controlled after a cure of hyperthyroidism but quickly returned to normal by one-month steroid therapy, implying MMI might be a trigger for AIH [11]. In this study, we reported a case of GD complicated with CHB and MMI-induced liver injury. Liver biopsy was performed on the 43rd day of hospitalization based on the withdrawal of MMI for eight weeks and effective inhibition of HBV replication for one month and thyroxine returning to normal for two weeks to minimize the influence of other factors (MMI toxicity, HBV replication and hyperthyroidism toxicity) on liver injury. AIH was then identified based on features of typical histology and immunology and good response to steroid, speculating MMI as a possible trigger for AIH. However, we can’t exclude the possibility of HBV as a trigger for AIH [12, 13].

**Table 2:** The differences of GD together with AIH

Year	Authors	Clinical features	Diagnosis	Therapy	Effect	Ref. no
1999	Inoue K, et al	A 48-year F suffered from GD and AIH concurrently. A11 and DR4 are positive in HLA	GD-associated AIH	MMI	fT4 and ALT were decreased simultaneously after MMI use	[19]
2011	Sato et al	A 58-year F had three-year GD, and then PTU-induced AI-DILI	GD and PTU- induced AI-DILI	PSL	Elevated AST level decreased immediately by PSL	[22]
2019	Sano et al	A 15-year F suffered from GD and treated with MMI. She was ill with MMI-induced DILI with AIH futures	GD and MMI- induced-DILI with AIH futures	RIA, PSL	Elevated aminotransferases couldn’t be controlled after RIA but normalized after one-month PSL	[11]
2019	Sano et al	A 21-year F suffered from GD complicated with classical type II AIH	GD and type II AIH	RIA, PSL, LT	AIH couldn’t be controlled by RIA and PSL, and received LT	[11]
2019	Sano et al	A 39-year-F suffered from GD complicated with classical AIH	GD and AIH	PSL, RIA, LT	AIH couldn’t be controlled by PSL and RIA, and received LT	[11]
2019	Sawhney et al	A 24-year F was ill with classical type I AIH, and then GD was found	Type I AIH and GD	PSL, AZA, RIA	AIH be effectively controlled by the combination of PSL and AZA and RIA	[20]

*F* female, *GD* graves’ disease, *AIH* autoimmune hepatitis, *AI-DILI* autoimmune-like drug-induced liver injury, *MMI* methimazole, *PTU* propylthiouracil, *PSL* prednisone, *RIA* radioactive iodine ablation, *LT* liver transplant, *AZA* azathioprine

The patient met the diagnosis for GD complicated with CHB and MMI-associated AI-DILI as well as an early stage of ACLF according to his clinical features after admission. Thus, a comprehensive treatment including HBV inhibition, sufficient nutrition, and hepatoprotective drugs and plasma was carried out immediately, which was reasonable. According to the guidelines for diagnosis and management of hyperthyroidism, the use of total/subtotal thyroidectomy was favored in such clinical situations as active Graves’ orbitopathy, patient with periodic paralysis, thyroid malignancy confirmed or suspected, and one of more large thyroid nodules [2]. Although the surgery was also accepted in the situations of coexisting with pregnancy or liver disease or major adverse reactions to anti-thyroid drugs, it was contraindicated in this patient due to liver failure. Therefore, radioactive ^131^I therapy became the best choice for hyperthyroidism in this case [2]. Since ^131^I therapy can damage thyroid acinar cells to increase the release of the thyroxine in about two weeks and aggravate liver failure, it was difficult to perform this procedure in ACLF. The patient underwent five DPMAS plus PE therapy within 18 days after admission, which sufficiently improved liver failure to be able to safely administer ^131^I treatment on the 10th day of hospitalization. However, serum bilirubin and ALT were still at high levels after effective control of HBV replication and hyperthyroidism. Glucocorticoids have become the first-line treatment for refractory AI-DILI or idiopathic AIH, with a short course of treatment and no recurrence for AI-DILI [1, 23]. It has been reported to be effective in PTU-induced AI-DILI [1]. In this case, AIH was completely cured by 3-month steroid therapy without recurrence. However, the exact cause of AIH needs to be further studied.

In summary, we report a case of GD overlapping with CHB and MMI-induced liver injury and AIH with an early stage of ACLF. We propose that the combination of CHB and MMI-induced liver injury and hepatotoxicity of excess thyroxine resulted in ACLF. AIH should be considered when ALT and bilirubin are still at high levels after stopping the culprit drug and gaining effective control of HBV replication and hyperthyroidism. Liver biopsy is helpful in the diagnosis of AIH. Glucocorticoids play an important role in the treatment of AIH.

## Data Availability

The datasets used during the current study are available from the corresponding author on reasonable request.

## Abbreviations

MMI: Methimazole
GD: Graves’ disease
ATP: Adenosine triphosphate
ROS: Reactive oxygen species
ANCA: Antineutrophil cytoplasmic antibody
IAS: Insulin autoimmune syndrome
CHB: Chronic hepatitis B
AI-DILI: Autoimmune-like liver injury
AST: Aspartate aminotransferase
ALT: Alanine aminotransferase
ALP: Alkaline phosphatase
TBil: Total bilirubin
CHE: Cholinesterase
Alb: Albumin
DBil: Direct bilirubin
TBA: Total bile acid
PTA: Prothrombin activity
INR: International normalized ratio
TSH: Thyroid stimulating hormone
FT3: Free triiodothyronine
ULN: Upper limit of normal
TgAb: Anti-thyroglobulin antibody
ANA: Antinuclear antibody
ACLF: Acute-on-chronic liver failure
ETV: Entecavir
DPMAS: Dual plasma molecular adsorption system
PE: Plasma exchange
UDCA: Ursodeoxycholic acid
HE: Hematoxylin–eosin
AECHB: Acute exacerbation of chronic hepatitis B
